# The Effect of Marine Derived *n*-3 Fatty Acids on Adipose Tissue Metabolism and Function

**DOI:** 10.3390/jcm5010003

**Published:** 2015-12-31

**Authors:** Marijana Todorčević, Leanne Hodson

**Affiliations:** Oxford Centre for Diabetes, Endocrinology and Metabolism, University of Oxford, Churchill Hospital, OX3 7LE Oxford, UK; marijana.todorcevic@ocdem.ox.ac.uk

**Keywords:** *n*-3 fatty acids, subcutaneous, adipose tissue, marine

## Abstract

Adipose tissue function is key determinant of metabolic health, with specific nutrients being suggested to play a role in tissue metabolism. One such group of nutrients are the *n*-3 fatty acids, specifically eicosapentaenoic acid (EPA; 20:5*n*-3) and docosahexaenoic acid (DHA; 22:6*n*-3). Results from studies where human, animal and cellular models have been utilised to investigate the effects of EPA and/or DHA on white adipose tissue/adipocytes suggest anti-obesity and anti-inflammatory effects. We review here evidence for these effects, specifically focusing on studies that provide some insight into metabolic pathways or processes. Of note, limited work has been undertaken investigating the effects of EPA and DHA on white adipose tissue in humans whilst more work has been undertaken using animal and cellular models. Taken together it would appear that EPA and DHA have a positive effect on lowering lipogenesis, increasing lipolysis and decreasing inflammation, all of which would be beneficial for adipose tissue biology. What remains to be elucidated is the duration and dose required to see a favourable effect of EPA and DHA *in vivo* in humans, across a range of adiposity.

## 1. Introduction

Adipose tissue, the largest organ in the human body, was historically considered to be metabolically inert. However, white adipose tissue is now considered an endocrine organ as it secretes adipokines (and hormones) which act locally and distally through autocrine, paracrine and endocrine effects [1]. Although adipose tissue is comprised of several cell types, including pre-adipocytes, adipocytes, endothelial cells, fibroblasts, leukocytes and macrophages [2], all of which may impact on tissue function, one of the main functions of adipocytes/adipose tissue is to store fatty acids [3]. Adipose tissue acts to “buffer” the influx of dietary fat into the circulation [3,4], with excess dietary fat being stored in adipose tissue rather than “overflowing” to non-adipose organs. Ectopic fat deposition has been proposed to underlie obesity-associated metabolic diseases [5]. An increase in adipose tissue mass may alter the function of the tissue. For example, when adipose tissue starts to expand (such as with excess nutrition) macrophages infiltrate and orchestrate inflammatory responses via molecules such as tumor necrosis factor α (TNFα), interleukin 6 (IL-6) and monocyte chemoattractant protein-1 (MCP-1), all of which have been implicated in the development of pathological changes in adipose tissue physiology [6,7,8,9]. Intriguingly, a proportion of overweight/obese individuals remain metabolically healthy even with further weight gain, whilst others do not; it has recently been suggested this is due to an increased capacity of adipose tissue for lipogenesis [10]. Multiple factors have been suggested to impact on the function of adipose tissue, however as the tissue is the primary site for dietary fat storage and reflects dietary fat intakes [11] it is reasonable to suggest that the composition or type of fat that the tissue is exposed to may also influence the function.

A class of fatty acids that has received a lot of attention over the last 30 years is the *n*-3 (or ω-3) fatty acids, specifically those derived from marine sources. *n*-3 fatty acids have been suggested to lower the risk of a number of non-communicable metabolic diseases including cardiovascular disease, obesity and diabetes [12,13,14]. Here we review the effect of long chain *n*-3 polyunsaturated fatty acids (LCPUFA), specifically eicosapentaenoic acid (EPA, 20:5*n*-3) and docosahexaenoic acid (DHA, 22:6*n*-3) on white adipose tissue metabolism and function. Although other *n*-3 fatty acids such as α-linolenic acid (ALA, 18:3*n*-3) and docosapentanoic acid (DPA, 22:5*n*-3) are of potential interest, data are limited. A number of reviews on the effect of fish oil or *n*-3 fatty acids on adipose tissue have previously been undertaken [15,16,17,18,19,20], therefore we have chosen to review the evidence from human, animal (rodent and fish) and *in vitro* cellular studies regarding the specific effects EPA and DHA have on the metabolism and function of white adipose tissue from different depots. Specifically, we will discuss the mechanisms by which EPA and DHA are proposed to reduce adiposity along with discussion regarding how *n*-3 fatty acids may influence markers of adipose tissue inflammation and cytokine production.

## 2. Dietary Sources of Eicosapentaenoic Acid (EPA) and Docosahexaenoic Acid (DHA)

EPA and DHA, commonly referred to as fish oil fatty acids, are not synthesized *de novo* by fish. Fish accumulate them through consumption of water plants, such as plankton and algae, which are part of the marine food chain [21]. Therefore, if plankton and algae are not a dietary component or if fish oil is replaced by other feed sources, such as in fish farming where a vegetable-oil based diet rich in linoleic acid (18:2*n*-6) and oleic acid (18:1*n*-9) may be given, the EPA and DHA content of the fish will decrease [22,23]. Marine fish tend to have higher amounts of EPA and DHA than freshwater fish. Fish typically store EPA and DHA mainly as triacylglycerol, at the middle position (sn-2) of the glycerol backbone however, in krill, a shrimp-like crustacean that feed off algae in deep ocean waters, 30%–65% of EPA and DHA is in phospholipids [24].

Within the human diet, EPA and DHA can be produced from ALA but the capacity of conversion is low in humans, although higher in women of child-bearing age than men [25]. Thus, it is likely that the majority of EPA and DHA within the body, for most individuals are derived from fish and fish oil intakes. Fish oil is often considered to be the best source of EPA and DHA however, as mentioned above, the amount of EPA and DHA varies amongst species and within a species according to environmental variables such as diet, temperature and salinity of the water.

## 3. Fatty Acid Composition of Adipose Tissue

As the fatty acid composition of adipose tissue has a half-life between 6 months and 2 years, it reflects long-term dietary intake along with endogenous metabolism [11]. The abundance of EPA and DHA in human subcutaneous adipose tissue is low, typically less than 0.2 for EPA and up to 1.0 mol% for DHA [11]. The amount of EPA and DHA in adipose tissue has been reported to increase or remain unchanged with increasing age [26,27,28], which is suggested to be an age-dependent effect independent of dietary intake [28].

Studies measuring the change in adipose tissue fatty acid composition, as a marker of compliance to *n*-3 supplementation are limited and findings inconsistent with some [29,30,31,32] but not all [33,34] noting small but significant increases in the abundance of adipose tissue EPA and DHA after varying periods of fish oil supplementation (Table 1). The inconsistency in findings may in part be explained by differences in: duration of supplementation, amount of EPA and DHA consumed, participant age, sex and adiposity, or site where the adipose biopsy was taken. Elegant work by Katan *et al.* [30] clearly demonstrated that the levels of DHA rose more rapidly in subcutaneous abdominal compared to gluteal adipose tissue depots whilst differences between the depots for EPA were not as obvious (Table 1). The difference in the appearance of DHA in subcutaneous abdominal compared to gluteal adipose tissue, may be explained by the fact that dietary fat extraction (from chylomicron-triacylglycerol) occurs to a greater extent in subcutaneous abdominal than gluteal adipose tissue [35]. Of note, Katan *et al.* [30] found that the proportion of EPA and DHA in subcutaneous abdominal and gluteal adipose tissue was approximately one-sixth and one-third respectively of dietary intake. It would be of interest to determine the extent to which the fatty acid composition of visceral (intra-abdominal) adipose tissue changed with *n*-3 fatty acid supplementation. However, as visceral adipose tissue samples are often obtained during elective surgery, it would be challenging to undertake a well-controlled study. Taken together, the data presented in Table 1 clearly demonstrate that even with supplementation the abundance of EPA and DHA in adipose tissue does not increase notably. This suggests that EPA and DHA are not preferentially stored in adipose tissue triacylglycerol long-term, rather they may be partitioned to oxidation pathways or to storage in other lipid fractions, such as phospholipids; red blood cell and plasma phospholipids have a notably higher abundance of both EPA and DHA than adipose tissue [11]. However, a change in adipose tissue fat mass and therefore dilution of EPA and DHA abundance cannot be ruled out as the majority of studies do not indicate if there were changes in participants’ body weight over the course of the study. Changes in fatty acid composition of adipose tissue have been reported with weight loss, notably there was not change in EPA abundance but an increase in DHA abundance, without a reported change in *n*-3 fatty acid intake, over the weight maintenance period [36]. These changes highlight the importance of weight/fat mass stability in subjects participating in intervention studies where adipose tissue fatty acid composition is being measured as a marker of compliance.

**Table 1:** Overview of human studies investigating change in eicosapentaenoic acid (EPA) and docosahexaenoic acid (DHA) abundance in subcutaneous adipose tissue.

Reference	Study Design	Subjects	Dose	Length	SCAT Biopsy Site	Abundance in AT EPA and DHA
[33]	Randomized double-blind, placebo controlled, parallel groups	Control: *n* = 25 (12 M/13 F) Age 55.4 y; BMI 29.5 kg/m^2^	Control: 2 g olive oil	6 wk	Gluteal	Control (baseline *vs.* 6 wk): EPA—0.11% to 0.11%; DHA—0.29% to 0.29%
		*n*-3 PUFA: *n* = 25 (12M/13F) Age 58.0 y, BMI 30.8 kg/m^2^	*n*-3 PUFA: 2 g fish oil/d (640 mg EPA and 480 mg DHA)			*n*-3 PUFA: EPA—0.12% to 0.13%; DHA—0.27% to 0.30%
[34]	Randomized double-blind, placebo controlled	Control: Pre-menopausal: *n* = 22 Age 44 y, BMI 24.6 kg/m^2^; Post-menopausal: *n* = 23 Age 55.6 y, BMI 23.1 kg/m^2^	Control: 4 g thistle oil	12 wk	Gluteal	Control (baseline *vs.* 12 wk): Pre-menopausal: EPA 0.1% to 0.1%; DHA 0.2% to 0.3%; Post-menopausal: EPA 0.1% to 0.1%; DHA 0.3% to 0.3%
		Fish oil: Pre-menopausal: *n* = 23 Age 41.6 y, BMI 24.5 kg/m^2^; Post-menopausal: *n* = 22, Age 56.0 y; BMI 24.5 kg/m^2^	Fish oil: 4 g fish oil (38.5% EPA and 25.9% DHA)			Fish oil: Pre-menopausal: EPA 0.1% to 0.1%; DHA 0.2% to 0.2%; Post-menopausal: EPA 0.1% to 0.2%; DHA 0.3% to 0.4%
[31]	Observational	Eight control	Control: low fish/no fish oil supplementation	12 m	Not reported	Control Group: EPA 0.003% (total FA); DHA 0.1%
		Seven patients attending lipid disorder clinic	Patients (fish oil): 10–15 g MaxEPA (17% EPA, 10.6% DHA)			Fish oil Group: EPA 0.4%; DHA 0.7%
[32]	Randomised placebo controlled parallel	Control: *n* = 14 (6M/8F) Age ^‡^ 62 y, BMI ^‡^ 29.2 kg/m^2^ all had T2D	Control: 20 g/d corn oil	9 wk	Gluteal	Control (0 *vs.* 9 wk); EPA 0.16% to 0.15%; DHA 0.39% to 0.39%
		Fish oil: *n* = 12 (7M/5F) Age ^‡^ 57 y, BMI ^‡^ 30.1 kg/m^2^ all had T2D	Fish oil: 20 g/d fish oil (13% EPA, 21% DHA)			Fish oil (0 *vs.* 9 wk); EPA 0.18% to 0.23%; DHA 0.49% to 0.55% *
[30]	Parallel study 4 groups (0, 3, 6 or 9 g fish oil/d)	58 months; Age 56.2 y	0 g/d = olive + palm oil; 3 g/d = 0.81 g EPA, 0.16 g DHA; 6 g/d = 1.62 g EPA, 0.33 g DHA; 9 g/d = 2.43 g EPA, 0.49 g DHA	12 m	AbdominalGluteal	Average change/g FA/d; EPA: Abdo = ↑0.12 wt %; Gluteal = ↑0.11 wt %
						DHA: Abdo = ↑0.24 wt %; Gluteal = ↑0.14 wt %
[29]	Parallel study 5 groups (received capsules to be equal to one portion of oily fish for 0, 1, 2 or 4 d/wk)	M and F 20–80 y; BMI >18 or <35 kg/m^2^	0 = high oleic sunflower oil; 1 = 1.5 g EPA, 1.77 g DHA/wk; 2 = 3.0 g EPA, 3.54 g DHA /wk; 4 = 6.0 g EPA, 7.08 g DHA/wk	12 m	Abdominal	Average change (% total FAs) compared to 0 portions; EPA: 0 portions = 0.18 % total; 1 portion = ↑0.05 % total; 2 portions = ↑0.04 % total; 4 portions = ↑0.11 % total **
						DHA: 0 portions = 0.22 % total; 1 portion = ↑0.05 % total; 2 portions = ↑0.06 % total; 4 portions = ↑0.13 % total **

Abbreviations: Ref, reference; M, males; F, females; y, years; BMI, body mass index; m, months; d, day; wk, week; T2D, type 2 diabetes; PUFA, polyunsaturated fatty acids; EPA, eicosapentaenoic acid; DHA, docosahexaenoic acid; SCAT, subcutaneous adipose tissue; FA, fatty acid; abdo, abdominal. Mean reported unless otherwise stated; ^‡^ median reported * *p* < 0.05 between baseline and end of study; ** *p* < 0.001 increase across groups.

## 4. The “Anti-Obesity” Effect of EPA and DHA

Measuring an anti-obesity effect of increased EPA and DHA consumption in humans is challenging, not least as there are many other factors to control for (e.g., exercise and other dietary components) and methodology sensitive to small changes in adipose tissue mass needs to be used. In 2009, Buckley and Howe [37] reviewed the available evidence for an anti-obesity effect of EPA and DHA. They suggested from the limited human studies, that increased consumption of EPA and DHA may reduce body fat; the majority of these studies were short-term, with a small number of subjects. It remains unclear if similar conclusions can be drawn from longer-term studies. A recent meta-analysis by Du and colleagues [38] identified randomised, placebo controlled trials where adults were assigned to either fish oil/marine group for a period of greater than 4 weeks and had reported at least one anthropometric measure of body composition (*i.e.*, body weight, BMI, waist circumference or waist to hip ratio). From the 21 studies (a total of 1329 individuals) they found no evidence to support an anti-obesity role of *n*-3 LCPUFA [38]. It is plausible that changes were not detected due to the non-specific and insensitive methods used to assess changes in body fat. By using computed tomography Sato *et al.* [39] noted that 6 months supplementation with EPA only (1800 mg/day) resulted in a significant decrease in epicardial and visceral adipose tissue mass, with no change in subcutaneous abdominal adipose tissue, in individuals with confirmed coronary artery disease. It is possible that subcutaneous abdominal adipose tissue mass did decrease however it was only measured in a single slice at the level of the umbilicus, thus changes in other depots would not have been detected. Results from some, but not all animal studies have suggested EPA and DHA consumption to have an anti-obesity effect with a lack of increase in fat mass even when an obesogenic diet is consumed [40], as well as a reduction in body weight if already obese [41]. Moreover, these studies, along with cellular studies have been used to tease out the mechanisms involved in this process, as discussed below with data from human [42,43,44,45,46,47,48], animal [40,41,49,50,51,52,53,54,55] and cellular [56,57,58,59,60,61,62,63,64,65,66,67,68,69] studies provided in Table 2, Table 3 and Table 4.

### 4.1. Suppression of Fat Deposition and Adipogenesis

A decrease in fatty acid deposition within adipose tissue may occur due to a decrease in triacylglycerol synthesis via decreased *de novo* lipogenesis or re-esterification of fatty acids within the tissue; alternatively it may occur due to a lower flux of fatty acids to the tissue. In the latter situation, fatty acids could be repartitioned to other tissues (such as muscle) for disposal, rather than going to adipose tissue for storage. In humans, the absolute contribution of *de novo* synthesized fatty acids to adipose tissue triacylglycerol is potentially small [70] and measuring adipose tissue *de novo* lipogenesis (or fatty acid esterification/re-esterification) *in vivo* in humans is challenging. Therefore, it is not surprising that studies have not been undertaken investigating how EPA and DHA supplementation influence these processes in humans. Although not a direct measure of fatty acid synthesis or esterification/re-esterification within the tissue, the measurement of the expression of genes related to these processes provides some insight to the effect of EPA and DHA on these processes. Camargo *et al.* [42] reported that consumption of 4 g/day of fish oil (containing a total of 1.24 g EPA and DHA) for 12 weeks significantly decreased the expression of genes related to fatty acid uptake and storage in subcutaneous obese adipose tissue (Table 2).

Work in animal models has typically found EPA and DHA to limit lipid accumulation in adipose tissue (Table 3). The majority of studies have reported lower fad pad mass and adipocytes number and size which was suggested to occur via suppression of lipogenic genes and, in some studies, a concomitant activation of lipolytic genes after supplementation with EPA and DHA (Table 3). Despite reporting a significant decrease in inguinal retroperitoneal fat pad mass Hainault *et al.* [52] did not find any significant change in fatty acid synthase (FAS) activity or mRNA expression in these depots. Of note, one study reported that EPA and DHA consumption resulted in higher total and perigonadal fat mass than control group [55]. This discrepancy in findings maybe in part explained that this study used an LDL receptor deficient (LDLR^-/-^) mouse model whilst others have typically used C57Bl/6 mice or Wistar rats.

**Table 2:** Overview of human studies investigating the effect of EPA and DHA supplementation on markers of adipose tissue metabolism and function.

Reference	Study Design	Subjects	Dose	Length	Measured	Adipose Tissue Outcome
[42]	Parallel (LIPGENE study) 4 Groups	Group 1. high SFA (*n* = 8, Age 57.8 y; BMI 36 kg/m^2^)	Group 1. No *n*-3	12 wk	SCAT abdo; mRNA expression of genes related to fatty acid uptake and storage	*n*-3 Supplementation group only had a significant decreased expression of PLIN1 and FABP4
		Group 2. high MUFA (*n* = 9, Age 57.1 y; BMI 34.5 kg/m^2^)	Group 2. No *n*-3			
		Group 3.LFHCC (plus 4 × 1 g/d sunflower oil) (*n* = 12, Age 56.5 y; BMI 35.7 kg/m^2^)	Group 3. supplement 4 × 1 g sunflower oil			
		Group 4. LFHCC plus 4 × 1 g/d FO (*n* = 10, Age 54.8 y; BMI 35.0 kg/m^2^)	Group 4. supplement 4 × 1 g FO (1.24 g *n*-3 fatty acids in ratio 1.4 EPA:1 DHA)			
[45]	Parallel (LIPGENE study) 4 Groups	See Reference [42] (Table 2) for participant characteristics and dietary groups	Group 1. No *n*-3	12 wk	SCAT abdo mRNA and protein expression of genes related to insulin signaling and carbohydrate metabolism	*n*-3 Supplementation for 12 wk increased expression of IRS-1 protein and CAP and decreased the expression of JNK, pAKT, EHD2, GAPDH, PEPCK1 and Anxa2. There was no change in PDK1
			Group 2. no *n*-3			
			Group 3. supplement 4 × 1 g sunflower oil			
			Group 4. supplement 4 × 1 g FO (1.24 g *n*-3 fatty acids in ratio 1.4 EPA:1 DHA)			
[47]	Parallel (LIPGENE study) 4 Groups	See Reference [42] (Table 2) for participant characteristics and dietary groups	Group 1. No *n*-3	12 wk	SCAT abdo mRNA expression of genes related to antioxidant processes; Postprandial = 4 h after high fat meal consumption	Postprandial increase in AT NADPH oxidase subunit p40^phox^ after 12 wk consumption *n*-3 fatty acids; Compared to SFA diet postprandial expression of SOD2, GPX4, TXN and KEAP1 were significantly lower whilst GPX3 and TXNRD1 were significantly higher
			Group 2. no *n*-3			
			Group 3. supplement 4 × 1 g sunflower oil			
			Group 4. supplement 4 × 1 g FO (1.24 g *n*-3 fatty acids in ratio 1.4 EPA:1 DHA)			
[46]	Parallel	Control (*n* = 13 (5M/8F) 37.8 y; BMI 30.1 kg/m^2^)	Control: *n*-3 fatty acids %TE intake = 0	14 wk (2 wk isocaloric, 12 wk *ad* *libitum*)	SCAT abdo; mRNa expression of gene related to inflammation	No change in the mRNA expression of genes encoding mediators of inflammation after consumption of *n*-3 fatty acids or when compared to control group.
		*n*-3 PUFA (*n* = 11 (3M/8M) 40.5 y; BMI 30.4 kg/m^2^)	*n*-3 PUFA: EPA—0.68% TE and DHA—0.47% TE			
[44]	Parallel	Control (*n* = 28 (15M/23F) 38 y; BMI 44.6 kg/m^2^	Control: butter fat (5g/d) on control diet	8 wk	VAT and SCAT abdo biopsies taken at end of intervention only. Expression of inflammatory related genes. Production of anti-inflammatory *n*-3 PUFA-derived eicosanoids.	Compared to control significant decreases in SCAT abdo on *n*-3 PUFA group for CCL2, CCL3, IL6, HIF-1A, TGFB1, CD40 and an increase in ADIPOQ
		*n*-3 PUFA (*n* = 27 (14M/23F) 39 y; BMI 48.7 kg/m^2^	*n*-3 PUFA: 4 g/d *n*-3 as ethyl esters (46% EPA and 38% DHA)			No differences in inflammatory genes in VAT. DHA-derived lipid mediators were more increased in VAT than in SAT.
[43]	Parallel (2 doses)	Group A: *n* = 6 (4 M) age 50.5 ± 10.8 y; BMI < 27 kg/m2 with CKD	Group A: 6 MaxEPA capsule/d (180 mg and 120 mg DHA per capsule)	10 wk	SCAT Abdo mRNA expression of genes related to inflammation	Group A: decreased mRNA expression of MMP9 and CD68 (baseline *vs.* 10 wk)
		Group B: *n* = 6 (2 M) age 50.2 ± 6.7 y; BMI < 27 kg/m2 with CKD	Group B: 12 MaxEPA capsule/d (180 mg and 120 mg DHA/capsule)			Group B: non-significant increase in MMP9 and CD68 (baseline *vs.* 10 wk)
[48]	Parallel	Placebo: *n* = 14 (5M) 53.3 ± 2.2 y; BMI 33.4 (27–43) kg/m2 with IR	Placebo: 4 g/d corn oil	12 wk	SCAT Abdo FAC, macrophages, capillaries, expression of inflammatory genes	Baseline *vs.* 12 weeks: Abundance of EPA and DHA in SCAT Abdo increased in FO group only
		Fish oil: *n* = 19 (6 M), 48.8 ± 2.3 (sem) y; BMI 33.4 (27–43) kg/m^2^ with IR	Fish oil (FO): 4 g/d EPA and DHA (Lovaza/Omacor)			Significant decrease in macrophages and crown like structures in tissue of FO group only; Significant decrease in mRNA expression of tissue MCP-1 and CD68 in FO group only

Data for age and BMI where data was available presented as mean ± sem. Abbreviations: Ref, reference number; M, males; F, females; y, years, BMI, body mass index; CKD, chronic kidney disease; CAD, coronary artery disease; SFA, saturated fat rich diet (16% total energy (TE)); MUFA, monounsaturated fat rich diet (20%TE); LFHCC, low-fat, high complex carbohydrate diet; PUFA, polyunsaturated fatty acids; FO, fish oil; EPA, eicosapentaenoic acid; DHA, docosahexaenoic acid; SCAT, white subcutaneous adipose tissue; Abdo, abdominal; VAT, visceral adipose tissue; AT, adipose tissue, IR, insulin resistance; MMP9, metalloprotease; CD68 phagocytic activity; FAC, fatty acid composition; MCP-1, macrophage chemoattractant protein 1; PLIN1, perilipin; FABP4, fatty acid binding protein-4; CAV1, caveolin; IRS-1, insulin receptor substrate-1; CAP, cbl-associated protein; JNK, jun *N*-terminal kinase; pAKT, phosphorylated v-akt murine thymoma viral oncogene homolog; EHD2, EH-domain containin-2; PDK1, 3-phosphoinositide-dependent protein kinase-1; GAPDH, glyceraldehyde-3-phosphate dehydrogenase; PEPCK1, phosphenolpyruvate carboxykinase-1; SOD2, superoxide dismutase 2; GPX, glutathione peroxidase; TXN, thioredoxine; TXNRD1, thioredoxin reductase 1; CCL2, chemokine (C-C motif) ligand 2; CCL3, chemokine (C-C motif) ligand 3; IL6, interleukin 6; HIF-1A, hypoxia-inducible factor 1-α; TGFB1, transforming growth factor β1; CD40, Cluster of differentiation 40; ADIPOQ; adiponectin.

**Table 3:** Overview of animal studies investigating the effect of EPA and DHA supplementation on markers of adipose tissue metabolism and function.

Reference	Study Design/Diet	Model	Dose	Duration	Measured	Adipose Tissue Outcome
[41]	Weight gain HF diet	C57BL/6 J mice	EPA and DHA combined increasing from 1% to 12% (*wt*/*wt*) dietary lipids	7–8 wk	Adiposity	AT accumulation limited when the amount of EPA/DHA increased on high fat diet. Epididymal fat decreased by 30%–50% of tissue cellularity.
[51]	HF diet with different combination of fatty acids added: 4 groups	4 m C57BL/6 J male mice	Group 1: HF-F high fat with 20% (*wt*/*wt*) flaxseed oil	4–5 wk	Adiposity	The EPA/DHA group (HF-F2) decreased body weight and had lowest increase in epididymal fat.
			Group 2: HF-F2: 44% dietary lipids—6% EPA and 51% DHA (EPAX1050)			
			Group 3: cHF-HF low *n*-3 PUFA content			Epididymal mRNA expression of genes related to OXPHOS and fatty acid uptake increased and those related to lipogenesis decreased.
			Group 4: HF-F1 high fat 15% EPAX1050			
[50]	HF diets comparing MaxEPA oil, herring oil, olive oil + beef tallow	50 d Wistar rats	MaxEPA—*n*-3 fatty acids ~41% diet; Herring oil—*n*-3 fatty acids-3 ~19% diet; Olive oil + beef tallow: *n*-3 fatty acids ~1% diet	4 wk	Adiposity	MaxEPA group has significantly lower lipid mass and fat cell size (but no change in number) in retroperitoneal fat compared to the low *n*-3 (olive oil + beef tallow) and herring oil diets.
						MaxEPA group had significantly lower epididymal fat mass and fat cell number compared to olive oil + beef tallow group.
[52]	HF (50% TE) diets. Three groups:	6 wk male Wistar rats	Not described	16–20 d	Adiposity	High lard and high lard plus corn oil significantly increased retroperitoneal fat whilst high lard plus FO had significant decrease in weight of inguinal, retroperitoneal and epididymal AT.
	Group 1. high lard					
	Group 2: high lard plus FO					No change in any group in FAS activity or expression in inguinal and retroperitoneal fat depots.
	Group 3: high lard plus corn oil					
[49]	HF feeding with corn oil or FO	Male Fisher 344 rats	40% diet FO or 40% diet corn oil	6 wk	Adiposity	FO group had significantly lower epididymal fat pads than the corn oil group.
[53]	HF feeding with or without FO	Male C57Bl/6 (WT) or GPR120 knockout mice—15 wk	With or without 27% menhaden FO (*wt*/*wt* menhanden FO 16% EPA and 9% DHA)	5 wk	AT inflammation	Wild-type animals: FO group had decreased mRNA expression of genes related to inflammation and macrophage infiltration in AT.
						FO supplementation had no effect in GPR120 knockout.
[55]	HF diets (39% energy) comparison of olive oil and FO.	LDL receptor deficient (LDLR^−/−^) mice on C57BL/6 J background. Females 2–3 m old.	Olive oil group: 6% energy olive	12 wk	Adiposity and inflammation	Compared to olive oil group the FO group had: - significantly higher total and perigonadal fat mass than olive oil group. - significantly higher distribution of larger adipocytes. - significantly increased AT cholesterol content and decreased gene expression in WAT related to inflammation and insulin sensitivity compared to olive oil group.
			FO group: 6% energy menhaden oil (140 mg EPA and 95 mg DHA/g oil)			
[54]	Control (FO 6% fat dry wt) and cafeteria (HF 62% fat dry wt) and	Male Wistar rats	Control and cafeteria groups: EPA 1 g/1kg/per day	5 wk	Adiposity, apoptosis and inflammation	Cafeteria + EPA group had lower fat mass gain, reduced retroperitoneal fat mass, decreased food intake and increased leptin production compared to cafeteria only fed rats. Control + EPA group had marked increase in markers of adipocyte apoptosis compared to control only. No different in cafeteria fed groups.
						TNFα expression significantly decreased in cafeteria + EPA compared to cafeteria only.
[40]	High and low dietary levels of EPA and DHA	Atlantic salmon	Control (rapeseed oil 10% of total fatty acids), FO (20% of total fatty acids), DHA enriched oil diet (42% DHA and 9% EPA), EPA enriched oil diet (43% EPA and 12% DHA)	21 wk	Lipid accumulation, β-oxidation, apoptosis	FO in decrease fat percentage of WAT and increase the FA β-oxidation capacity.
						High levels of DHA and EPA in DHA and E PA enriched oil diets lead to, loss of mitochondrial functions, and induction of caspase-3, indicating an onset of apoptosis.

Abbreviations: Ref, reference number; EPA, eicosapentanoeic acid; DHA, docosahexanoic acid; wk, weeks, m, months; AT, adipose tissue; WAT, white adipose tissue; HF, high fat; FO, fish oil; FAS, fatty acid synthase; WT, wild-type; GPR120. G-couple protein receptor 120; LDL, low density lipoprotein; wt, weight; TNFα, tumor necrosis factor α.

Studies investigating the effects of dietary EPA and DHA on adipose tissue function have also been undertaken in fish (Table 3). Todorcevic *et al.* [40] demonstrated that a diet supplemented with EPA and DHA (20% of total fatty acids) for 21 weeks repressed the development of adiposity, regulating triacylglycerol accumulation in visceral adipose tissue of Atlantic salmon. A positive influence of dietary EPA and DHA on lipid accumulation in adipose tissue was also reported in grass carp [71]. Diet containing EPA and DHA, (12% of total fatty acids for 75 days), suppressed lipid accumulation in intraperitoneal adipose tissue and significantly up-regulated the expression lipolytic genes including: lipoprotein lipase (LPL), stearoyl-CoA desaturase 1 (SCD1) and peroxisome proliferator activated receptor α (PPARα) [71]. Furthermore, similar results were reported by Liu *et al.* [72] in grass carp treated with dietary EPA and DHA (11% of total fatty acids) for 95 days.

The process of adipogenesis (or an increase in fat mass) involves the differentiation of preadipocytes to mature adipocytes, is a complex and tightly regulated process involving a cascade of transcription factors which are sensitive to the nutritional environment [73]. In a comprehensive review by McMillen and Robinson [74] the role of the nutritional environment an individual is exposed to before birth and in early infancy impacts on risk of obesity and obesity-related diseases later in life was discussed. Evidence from animal studies shows that offspring of mothers fed a diet high in calories or high in fat before birth are heavier and have a higher percentage body fat throughout life [75,76]. Findings from human studies are compelling; children born to mothers who are obese during their pregnancy have an increased incidence of obesity over the life course [75]. Therefore, it has been suggested that targeting maternal nutrition during pregnancy may reduce risk of obesity in subsequent generations [77]; *n*-3 fatty acids may decrease adipogenesis and lipogenesis and thus exposure in utero to these fatty acids may lower the risk of obesity in offspring. In 2011 Muhlhausler and colleagues [77] reviewed animal studies to determine the effects of *n*-3 LCPUFA supplementation during pregnancy and lactation on postnatal body composition of offspring. Although 13 potential studies were identified, only four met the inclusion criteria and the authors found from albeit limited data that there was a suggestion that the offspring from *n*-3 LCPUFA supplemented dams had a lower fat mass [77]. In contrast, supplementation of dams with a high DHA diet (5% fat of which DHA was 0.95% total fatty acids) during pregnancy and lactation resulted in offspring that had significantly higher total and subcutaneous fat mass (as percentage of total body weight) at 6 weeks of age, compared to control animals fed a diet containing the same amount of fat but devoid of *n*-3 LCPUFA [78]. Thus it remains unclear if increased exposure in utero to *n*-3 fatty acids decreases adipogenesis and lipogenesis and is an area that warrants further investigation.

To date, the majority of *in vitro* evidence regarding the mechanistic effects of EPA and DHA on triacylglycerol accumulation/lipid deposition comes from the clonal murine cell line, 3T3-L1 (Table 4). This cell line offers advantages over primary cells, as they are homogenous with regards to cellular population and stage of differentiation; however, their ability to reflect human adipose tissue function and metabolism remains to be clarified. Primary pre-adipocyte cultures have been shown to better reflect *in vivo* adipose function, than cell lines because they can be isolated from different species and fat depots. The latter is of interest as there are distinct molecular and biochemical hallmarks between different adipose tissue depots and at a cellular level; pre-adipocytes isolated from different adipose tissue depots and cultured *in vitro* retain depot-specific functional properties [79,80,81]. Unlike cell lines, the function and metabolism of primary cells will be influenced by the age, sex, and genetics of the donor and therefore consideration is needed when comparing across studies.

Results from *in vitro* cellular studies that have added EPA and DHA to media for periods of 24 h to 3 weeks are mixed; some suggest EPA and DHA to be anti-adipogenic whilst others find a pro-adipogenic response. EPA and DHA have been found to inhibit, promote or have no effect on the differentiation of pre-adipocytes (Table 4). Typically the markers of adipocytes adipogenesis that have been measured include: the accumulation of triacylglycerol, lipid droplet formation, expression of master adipogenic transcription factors, and lipid genes. Using 3T3-L1 pre-adipocytes, Kim *et al.* [59] investigated the effects of DHA alone (6 days) on lipogenesis and lipolysis and found mean lipid droplet size, percent lipid area, as well as glycerol-3-phosphate dehydrogenase (GPDH) activity all significantly decreased whilst basal lipolysis increased in fully differentiated adipocytes. The results from this work demonstrate the anti-adipogenic effects of DHA via inhibition of triacylglycerol accumulation and increased lipolysis [59]. When comparing the effects of EPA and DHA on lipid droplet formation in 3T3-L1 cells it was found that although both fatty acids reduced the presence of lipid droplets, DHA was more potent than EPA [56]. In addition to the decreased lipid droplet formation, there were notable reductions in the expression of key protein involved in this process, including perilipin A, caveolin-1 and Cidea [56], however there was no effect of DHA on PPARγ expression [56]. In contrast, Murali *et al.* [62] reported that incubating 3T3-L1 with EPA and DHA induced adipogenesis; DHA being more potent than EPA in inducing the differentiation process. The authors suggested the differential effects of EPA and DHA on adipognesis could be due to differential accumulation of *n*-3 fatty acids in membrane phospholipids [62]. In line with Murali *et al.* [62], Wojcik *et al.* [69] reported increased accumulation of neutral lipids in mature 3T3-L1 adipocytes; however, others have reported no effect on triacylglycerol accumulation at any stage of maturation in 3T3-L1 adipocytes [65]. A reduced expression of both adipogenic and lipogenic genes, including sterol regulatory element-binding protein 1 (SREBP1), FAS, and peroxisome proliferator-activated receptor γ (PPARγ) after EPA and DHA treatment of mature adipocytes has been report by some [69] but not by others [67]. Using human breast adipocytes as a cell model, Wang *et al.* [68] demonstrated exposure of DHA for 24 h decreased the expression of lipogenic genes, including FAS, LPL and PPARγ, whilst expression of lipolytic genes was increased. Lee *et al.* [82] found EPA to stimulate glycerol and free fatty acids release which was associated with induction of lipolytic gene expression and suppression of adipogenic gene expression in 3T3-L1 adipocytes. Treatment of fish primary adipocytes with EPA and DHA (for 3 weeks) resulted in decreased triacylglycerol accumulation in mature adipocytes [83]. In an acute study, using mature adipocytes isolated from grass carp Liu *et al.* [72] found that 6 h of incubation with EPA and DHA was sufficient to notably decreased triacylglycerol accumulation, significantly increased glycerol release and the expression of genes involved in lipolysis (e.g., adipose triglyceride lipase (ATGL), hormone-sensitive lipase (HSL)). The findings from *in vitro* cellular studies, notably those using primary adipocytes demonstrate that EPA and DHA inhibit triacylglycerol accumulation, which may be the result of effects mediated through genes related to lipogenesis and lipolysis.

Overall, the effects of EPA and DHA as well as EPA *versus* DHA in modifying adipogensis and lipid accumulation, in particular in humans, and to a lesser extent in murine models, remain unclear. Plausible reasons the discrepancies in findings between *in vitro* cellular studies are likely to be in part due to the use of different *in vitro* models, *i.e.*, using primary cells *versus* immortalized cell lines, studying the cells at different developmental stages, differences in the concentrations of fatty acid(s) the cells were exposed to, along with the duration of exposure.

**Table 4:** Overview of cellular studies investigating the effect of EPA and DHA supplementation on markers of adipocytes metabolism and function.

Reference	Cell Type	Cell Stage	Control Cells *	EPA/DHA Dose	Culture Duration	Measured	Outcome
[59]	3T3-L1	Pre-confluent pre-adipocytes; Post confluent pre-adipocytes; Early and fully differentiated adipocytes	BSA	DHA: 25, 50, and 200 μM	4, 24, 48 h, and 6 d	DNA denaturation; lipid accumulation; GPDH and LDH activity; glycerol secretion in media	DHA had anti-adipogenic effect with decreased mean lipid droplet size and % of lipid area but increased basal lipolysis and apoptosis
[69]	3T3-L1	Different stages of differentiation	NI	EPA, DHA: 100 µM	24–48 h	Lipid accumulation; UPS activity; MTT cytotoxicity assay; expression of NFκB, TNFα, adiponectin, SREBP1, FAS, PPARγ	EPA and DHA reduced expression of adipogenic genes, decreased activity of UPS, increased accumulation of neutral fats and induced TNFα mRNA level
[67]	3T3-L1	Fully differentiated adipocytes	BSA	EPA, DHA: 100 µM	48 h	Expression of PPARγ, ACC1, SCD1, adiponectin	DHA did not affect expression of any measured genes.
							EPA only increased mRNA expression of SCD1
[61]	3T3-L1	Fully differentiated	DMSO and/or Ethanol	EPA: 100, 200 µM	24 h	Apelin secretion and gene expression	EPA stimulated apelin secretion and apelin gene expression
[57]	3T3-L1	Fully differentiated	TZD	EPA, DHA: 100 µM	48 h	Adiponectin secretion	EPA and DHA increased adiponectin secretion
[56]	3T3-L1	Fully differentiated	2% BSA + 100% ethanol	EPA, DHA: 100 µM	7 d	Lipid accumulation, glycerol realise in media and mRNA expression of adipogenic, lipolytic and LD markers	EPA and DHA reduced lipid droplet formation and SCD1 expression compared to cells treated with stearic acid.DHA increased lipolysis, ATGL gene and protein expression and reduced gene expression of perlipin, caveolin-1, Cidea
[60]	3T3-L1	Differentiated adipocytes	BSA	EPA, DHA: 100 µM	24 h	mRNA and protein levels of anti-oxidative enzyme HO-1, gene expression of SOD, CAT and GPX	EPA and DHA prevented oxidative stress induced HO-1 and activation of Nrf-2
[58]	3T3-L1	Differentiated adipocytes	NI	EPA: 100 µM	24 h	CPT-1—Activity, protein level and mRNA expression	EPA increased β-oxidation but did not inhibit lipogenesis
[62]	3T3-L1	Fully differentiated	Differentiation media no FA added	EPA, DHA: 50 µM	7 d	mRNA expression of PPARγ, C/EBPα, aP2; oil red O staining; adiponectin secretion; pro-inflammatory signalling pathways	DHA but not EPA significantly increased differentiation markers. DHA more effective than EPA at increasing adiponectin secretion. DHA only inhibited activation of ERK 1/2 and P38 MAPK
[65]	3T3-L1	Different stages of differentiation	Albumin	EPA: 100 μM; DHA: 50 μM	48 h	Lipid accumulation and glycerol release. Secretion of IL-6, leptin, adiponectin	EPA and DHA did not affect lipid accumulation or lipolysis. EPA and DHA increased secretion of adiponectin in early differentiated adipocytes. EPA and DHA had an opposite effect on IL-6 secretion: EPA increased secretion at all stages, DHA decreased it. EPA only had an impact on leptin secretion in early stage of differentiation
[64]	3T3-L1	Fully differentiated	Albumin	EPA, DHA: 125 μM	24 h	Adiponectin secretion and adiponectin cellular protein	EPA and DHA increased the secreted adiponectin concentration but did not affect cellular adiponectin protein content
[68]	Human breast adipocytes	Fully differentiated	NI	DHA: 50, 100 µM	24 h	mRNA expression of IL-6, TNFα, PPARγ, PPARα, HSL, perlipin, LPL, FAS, glycerol release	DHA decreased the expression of PPARγ and other lipogenic genes and increased the expression of lipolytic genes and glycerol release
[63]	Human primary adipocytes	Fully differentiated	Differentiation media or BSA	EPA, DHA: 5 and 10 μM	6 and 12 h	IL-6,TNFα, MCP1 secretion before and after LPS treatment	EPA and DHA reduced the secretion of LPS induced cytokine secretion
[66]	Human primary adipocytes	Fully differentiated	BSA	EPA, DHA: 100 µM	48 h	Adiponectin secretion and adiponectin cellular protein	EPA and DHA increased adiponectin secretion. EPA but not DHA increased cellular adiponectin protein

* Control or comparison cells. Abbreviations: EPA, eicosapentaenoic acid; DHA, docosahexaenoic acid; BSA, bovine serum albumin; h, hour; d, day; NI, not indicated; FA, fatty acid; GPDH, glycerol-3-phosphate dehydrogenase; LDH; lactate dehydrogenase; UPS, ubiquitin–proteasome system: NFκB, nuclear factor kappa-light-chain-enhancer of activated B cells; TNFα, tumor necrosis factor α; SREBP1, sterol regulatory element-binding protein 1; FAS, fatty acid synthase; PPARγ, peroxisome proliferator-activated receptor γ; TZD, troglitazone; SCD1, steroyl-CoA desaturase 1; ATGL, adipose triglyceride lipase; HO-1, heme oxygenase 1; Nef-2, Nucleotide Excision Repair Factor 2; LD, lipid droplet; SOD, Superoxide dismutase; CAT, catalyse; GPX, glutathione peroxidase; CPT-1, carnitine palmitoyltransferase 1; aP2, adipocyte protein 2; IL6, interleukin 6; HSL, hormone sensitive lipase; LPL, lipoprotein lipase; MCP-1, monocyte chemoattractant protein-1; LPS, Lipopolysaccharide; ERK1/2, extracellular-signal-regulated kinases; MAPK, Mitogen-activated protein kinases; MTT, colorimetric assay for assessing cell metabolic activity; ACC1, Acetyl-CoA carboxylase; DMSO, dimethyl sulfoxide.

### 4.2. Adipocyte Apoptosis

To our knowledge, there have been no studies in humans investigating the effect of *n*-3 fatty acids on adipocyte apoptosis and only limited work has been undertaken in animal and *in vitro* cellular models. Although outside the scope of this review, there have been a large number of studies investigating the effect of *n*-3 fatty acids and cancer in relation to apoptosis, as reviewed by Wendel and Heller [84].

Limiting findings from human *in vitro* and *in vivo* studies have reported apoptosis in white adipose tissue along with alternations in adipose tissue mass. Thus consideration is required when looking at adipose tissue mass in relation to cell number as they might be partly regulated by pre-adipocyte/adipocyte apoptosis [85,86]. Nelson-Dooley *et al.* [87] have suggested targeting apoptotic pathways in adipocytes as a novel way of treating obesity. Apoptosis is often assessed by cytomorphological alterations, DNA fragmentation and condensation, detection of caspases, protein cleavage at specific locations, cell membrane alterations and and increased mitochondrial membrane permeability [88]. In 2004 Ruszickova *et al.* [41] were the first to suggest the concept of *n*-3 fatty acids and regulation of cellularity in adipose tissue. Using a rodent model the authors suggested that increased intakes of EPA and DHA (Table 3) reduced high-fat diet-induced obesity by decreasing the number of adipocytes in adipose tissue, which could be interpreted as evidence of a pro-apoptotic effect. Perez-Matute *et al.* [54] demonstrated increased levels of histone-associated DNA oligonucleosomal fragments, classical markers of apoptosis in the white adipose tissue of rats fed a standard diet with additional oral administration EPA ethyl ester (1 g/kg per day) daily for 5 weeks. Moreover, they found a cafeteria diet strongly impaired the apoptotic action induced by EPA and suggested that EPA-induced apoptosis depends on the nutritional and metabolic status of the animals [54]. High dietary-*n*-3 fatty acid levels are at increased susceptibility to fatty acid peroxidation which has been reported to occur in different tissues within a fish model [89] including adipose tissue [40]. Fish contain a greater amount of more highly unsaturated fatty acids than mammals which makes them more prone to fatty acid peroxidation leading to apoptosis [90]. Todorcevic *et al.* [40] were the first to demonstrate that high dietary intakes of EPA and DHA induced oxidative stress and apoptosis in the visceral adipose tissue in Atlantic salmon. Salmon was fed with diets containing 50% EPA and 55% DHA of total fatty acid for 21 weeks and found increased activity of caspase-3, indicative of apoptosis occurring in white adipose tissue. The authors concluded that decreased adipocytes cell number due to apoptosis, may be one factor explaining the lower triacylglycerol accumulation occurring in fish white adipose tissue when diets enriched with EPA and DHA are fed [40]. On the basis of these finding, it would be prudent to suggest the measurement of adipose tissue apoptotic markers when EPA and DHA, notably at high dietary doses, are given.

Even though there is a growing literature on the studying the mechanisms for the inhibitory effects of *n*-3 fatty acids on proliferation of various tumor cells (reviewed by [91]) but also on non-cancerous cells [92], there are surprisingly very few *in vitro* studies that have investigated the effect of EPA and DHA on adipocyte apoptosis. Kim *et al.* [59] reported significant DHA-induced apoptosis in 3T3-L1 post-confluent pre-adipocytes after 48 h incubation with 200 µM/L compared to 100 µM/L DHA, demonstrating the inhibitory effects of DHA on adipocyte differentiation. Todorcevic *et al.* [93] treated primary antioxidant glutathione (GSH) depleted salmon adipocytes with high doses of EPA and DHA (600 µM for 6 days) in present or absence of α tocopherol and showed increased expression of genes encoding a set of well-known apoptotic markers in the groups with no added α tocopherol, suggesting that the induction of adipocyte cell death by EPA and DHA likely plays an important part in the adipose tissue homeostasis especially in animals exposed to high dietary *n*-3 fatty acids.

Taken together, the available data from animal and *in vitro* studies suggests that high doses of EPA and DHA may induce adipocyte apoptosis. How targeting the apoptotic pathway in white adipose tissue would decrease obesity and influence adipose tissue function and overall metabolic health in humans remains to be elucidated.

### 4.3. Increased Fatty acid Oxidation (Energy Expenditure)

Although an increase in fatty acid oxidation, via β-oxidation has been suggested to play a role in a reduction of triacylglycerol accumulation in adipocytes, evidence for this in white adipose tissue is limited; fatty acid oxidation and mitochondrial function has been studied more often in brown adipose tissue. The number and activity of mitochondria within adipocytes has been suggested to contribute to insulin resistance and type 2 diabetes [94]. Changes in the expression of genes related to insulin-signaling have been reported to increase, whilst the expression of genes related to glycolysis, gluconeogenesis and glyceroneogenesis decreased in subcutaneous abdominal adipose tissue after 12 weeks supplementation with *n*-3 fatty acids [45] (Table 2). On the basis of these changes, the authors suggested that a low-fat (fat 28% total energy (TE)) high complex carbohydrate diet supplemented with 1.24 g/day *n*-3 fatty acids (EPA and DHA) improved adipose tissue insulin sensitivity compared to diets high saturated or monounsaturated fat in individuals with the metabolic syndrome [45]. To lower the risk of obesity-mediated diseases such as the metabolic syndrome, weight loss is often encouraged to decrease fat mass; weight loss by calorie restriction has been suggested to increase subcutaneous abdominal adipose tissue capacity for lipid oxidation [95]. Whether similar changes occur in subcutaneous gluteal or visceral adipose tissue remains to be determined. Moreover, it would be of interest to determine if calorie restriction in combination with EPA and DHA supplementation has an additive effect on up-regulating adipose tissue fatty acid oxidation in different adipose tissue depots. The amount of EPA and DHA has varied between studies, with higher doses tending to be used in animal and *in vitro* studies, translation to the appropriate dose, along with duration required to see an effect in humans needs to be elucidated.

*In vivo* or *in vitro* cellular studies investigating the effects of EPA and DHA on adipose tissue fatty acid oxidation and/or mitochondrial function are sparse. Specifically measuring markers of adipose tissue fatty acid oxidation *in vivo* in humans has not, to our knowledge, been undertaken. This is most likely to be due to the challenges associated with assessing adipose tissue fatty acid β-oxidation directly. Surprisingly no study in humans has yet investigated changes in the expression of relevant genes in adipose tissue before and after supplementation with EPA and DHA. Fasting whole-body fatty acid oxidation (assessed by indirect calorimetry) has been reported to increase in young, healthy men (*n* = 5) after 3 weeks of supplementation with fish oil (6 g/day) when compared to a control diet containing equal amounts of total dietary fat [96]. Only a few animal studies have investigated the effect of EPA and DHA on fatty acid β-oxidation in white adipose tissue (Table 3). Flachs *et al*. [51] reported that feeding mice for 4 weeks with diet containing increasing amounts of EPA and DHA, preferentially up-regulated several mitochondrial regulatory genes, increased β-oxidation and suppressed lipogenesis in white abdominal fat. Using a fish model, Atlantic salmon, Todorcevic *et al.* [40] reported an increase in adipose tissue fatty acid β-oxidation after fish consumed fish oil rich in EPA and DHA for 21 weeks.

*In vitro* cellular studies have found increased β-oxidation in 3T3-L1 adipocytes after incubation with 100 µM of EPA for 24 h [58]. The increase in β-oxidation was associated with increased carnitine palmitoyltransferase 1 (CPT-1) activity but mRNA and protein expression did not change [58]. As EPA treatment increased the proportion of EPA in mitochondrial membrane lipids, the authors concluded that the activity of CPT-1 and β-oxidation was due to changes in the structure or dynamics of the mitochondrial membranes [58]. EPA and DHA are reported to activate AMP-activated protein kinase (AMPK) in 3T3-L1 adipocytes, which could be a mechanism for their effect on fatty acid oxidation [97]. Todorcevic *et al.* [83] demonstrated that EPA and DHA increased β-oxidation in salmon primary adipocytes, which may in part explain the concomitant reduction in adipocyte triacylglycerol. A possible mechanism, by which EPA and DHA may result in increased fatty acid oxidation and therefore less body fat accumulation, is through induction of thermogenesis mediated by mitochondrial uncoupling protein-1 (UCP1); the thermogenic capacity of brown adipose tissue (BAT) is associated with uncoupling whereas white adipose tissue is typically not [98]. In 2013 Flach *et al.* [98] reviewed the effect of *n*-3 fatty acids on mitochondrial oxidative phosphorylation (OXPHOS) and fatty acid oxidation in white adipose tissue. In this comprehensive review they reported that in a murine model, supplementation with *n*-3 fatty acids in combination with mild calorie restriction induced mitochondrial OXPHOS in epididymal white adipose tissue only, independent of UCP1 induction; other studies in rodents have reported increased levels of UCP1 mRNA and/or protein in BAT in response to *n*-3 fatty acid supplementation [98]. Recently, Zhao and Chen [99] using an *in vitro* cellular model of isolated stromal-vascular (SV) cells from inguinal adipose tissue of suggested that EPA enhanced energy dissipation capacity by recruiting brite adipocytes to stimulate oxidative metabolism. From the limited data available it appears that EPA and DHA increase fatty acid β-oxidation in adipocytes, however the mechanisms responsible and the effect on mitochondrial OXPHOS and thermogenesis in human adipose tissue remains to be elucidated.

## 5. The “Anti-Inflammatory” Effects of EPA and DHA on Adipose Tissue

An expansion of adipose tissue mass, is often associated with macrophage infiltration which may lead to inflammatory responses, which have been implicated in the development of pathological changes in adipose tissue physiology [6,7,8,9]. These changes potentially move the tissue toward a pro-inflammatory phenotype and there is accumulating evidence suggesting pro-inflammatory processes in adipose tissue increase the risk of obesity-related disorders, such as insulin resistance [100,101,102,103]. For example, several studies reported positive associations between degree of obesity and the expression of genes related to inflammation in adipose tissue [7,9]. A number of studies have investigated the “anti-inflammatory” effect of EPA and DHA in white adipose tissue.

### Suppression of Pro-Inflammatory Cytokine Production

Studies investigating the effect of *n*-3 fatty acid supplementation, for periods between 8 weeks up to 6 months, on the expression of genes related to inflammation in human subcutaneous white adipose tissue have been undertaken. Overall results are variable, with some suggesting consumption of EPA and DHA decreases the expression of genes related to inflammation, whilst other report no change (Table 2). For example, Guebre-Egziabher *et al.* [43] noted decreased expression of metalloprotease 9 (MMP9) and CD68 in subcutaneous abdominal adipose tissue on a low not high dose of MaxEPA in a small number (*n* = 12) of individuals with chronic kidney disease (CKD) who were randomised to take either a low (*n* = 6) or high (*n* = 6) dose of MaxEPA for 10 weeks. In contrast, Itariu *et al.* [44] found that high doses of EPA and DHA (total 4 g/day) for 8 weeks significantly decreased the expression of genes related to inflammation in subcutaneous obese adipose tissue and increased production of anti-inflammatory eicosanoids in visceral adipose tissue (Table 2).

Work in murine models has found consumption of *n*-3 fatty acids decreased inflammatory gene expression in white adipose tissue depots (Table 3). Todoric *et al.* [104] investigated the effect of an *n*-3 fatty acid diet on macrophage infiltration in white adipose tissue of obese, diabetic mice, as well as on gene expression of several immune genes. They found that consumption of 25.1 mg of *n*-3 fatty acids (containing EPA and DHA) per gram of fat for 6 weeks resulted in a reduction in macrophage infiltration in combination with decreased expression of inflammatory genes in white adipose tissue [104]. Sarawathi *et al.* [55] used LDLR^−/−^ mice and showed similar results to Todoric *et al.* [104] despite a gain in white adipose tissue mass. They reported a diet supplemented with fish oil containing 140 mg EPA and 95 mg DHA/day for 12 weeks reduced expression of macrophage markers such as MAC-1 and CD68 as well as inflammatory markers such as TNF*α*, metalloprotease 3 (MMP3), and serum amyloid A3 (SAA3) in white adipose tissue [55]. Taken together these data demonstrate that consumption of *n*-3 fatty acids have the potential to modulate immune response in adipose tissue.

*In vitro* studies, using cell-lines and human primary cells, have been utilised to investigate the potential cellular mechanisms and pathways involved in an *n*-3 fatty acid mediated alteration in immune response (Table 4). Adiponectin, an adipocyte-specific protein, is often suggested to be anti-inflammatory cytokine and it has been postulated that a change in secretion may be associated with visceral obesity [105]. *In vitro* cellular work has found that incubation of primary human adipocytes isolated from subcutaneous adipose tissue with either with EPA or DHA significantly increased the concentration of secreted adiponectin [106], which is in agreement with several studies performed using primary cultured rat adipocytes [107], 3T3-L1 adipocytes [64] and human adipocyte cell lines [66]. From the work of Oster *et al.* [64] it appears that EPA and DHA have differential effects on adiponectin secretion, which may be influenced by the cell model used. They found DHA increased adiponectin mRNA expression and secreted adiponectin protein to a greater extent than the same dose of EPA in 3T3-L1 adipocyte after 24 h incubation [64]. In contrast, Tishinsky *et al.* [99] found using a commercial line of human adipocytes that EPA significantly increased cellular adiponectin protein content after 48 h of treatment while DHA did not affect cellular adiponectin protein.

The effects of *n*-3 fatty acids on the adipokine leptin, have been investigated *in vitro* however results show conflicting effects of *n*-3 fatty acids on leptin mRNA expression and secretion. EPA has been shown to have a stimulatory effect on leptin gene expression and secretion in 3T3-L1 adipocytes [108] and primary cultured rat adipocytes [109]. Reseland *et al.* [110] reported an opposite effect to the work of Murata *et al.* [108] and Perez-Matute *et al.* [109], where exposure to both EPA and DHA reduced leptin mRNA expression in 3T3-L1 adipocytes. Furthermore, the effect of EPA and DHA on leptin expression has been shown to vary depending on the stage of adipocyte maturation [65]. Thus, the discrepancy in reported results could be related to differences in different cell models used (primary cells *vs.* cell lines) or in measuring the effects of *n*-3 fatty acids on leptin at different stages of adipogenesis. Culturing human primary adipocytes in either EPA or DHA resulted in a down-regulation of IL6 and TNFα secretion [63]. In contrast, differential effects of EPA and DHA were found for IL6 secretion in 3T3-L1 cells with EPA increasing and DHA decreasing secretion [65]; the underlying mechanisms for these responses were unable to be clarified by the authors. Another divergent finding is that from Wojcik *et al.* [69] who noted culturing 3T3-L1 cells in either EPA or DHA increased TNFα mRNA expression; it is unclear if this lead to increased secretion as it was not measured. The authors speculated that their finding would not be replicated in adipose tissue *in vivo*, as the anti-inflammatory effects of EPA and DHA on TNFα expression would be modulated through the direct effect of these fatty acids on macrophages; cells that were not present in their *in vitro* culture [69]. It remains unclear if EPA and DHA have a differential effect on anti-inflammatory markers in human and animal models as typically these fatty acids have been given together and not directly compared.

## 6. Conclusions

In recent years evidence demonstrating that an increased consumption of EPA and DHA may have a beneficial effect on white adipose tissue function and metabolism is starting to emerge. Although current literature cannot support an exact mechanistic role of EPA and DHA on adipose tissue biology it is apparent that these fatty acids have the potential to be potent modulators of adipose tissue and adipocyte function. More work has been undertaken using animal and cell models therefore consideration is required regarding the dose and duration of EPA and DHA, the animal and cell model used (e.g., primary *vs.* cell-lines). Moreover, *in vitro* cellular cells often investigate the effects of EPA and DHA on adipocytes and it is plausible a different response may be found in whole adipose tissue due to the presence of other cell types (e.g., macrophages, endothelial cells) and their interaction with adipocytes. Although the effects of *n*-3 fatty acid supplementation on the fatty acid composition of subcutaneous abdominal and gluteal adipose tissue have been investigated, mechanistic studies (*in vivo* and *in vitro*) appears to be limited to primarily subcutaneous abdominal adipose tissue and/or adipocytes. Evidence for an effect of *n*-3 fatty acids in human visceral adipose tissue is sparse and therefore not well understood. Evidence for a reduction in fat accumulation in animal models, along with an anti-inflammatory effect appears to be consistent when intakes of EPA and DHA are high (up to 20% of total fatty acids); however recommendations for human intakes are between 0.5% and 2% of total energy intake [111]. Thus, the duration and amount of dietary EPA or DHA required for beneficial effects in human subcutaneous adipose tissue depots remains to be elucidated.

## References

[1] Mohamed-Ali V., Pinkney J.H., Coppack S.W. (1998). Adipose tissue as an endocrine and paracrine organ. Int. J. Obes. Relat. Metab. Disord..

[2] Trayhurn P. (2007). Adipocyte biology. Obes. Rev..

[3] Frayn K.N. (2002). Adipose tissue as a buffer for daily lipid flux. Diabetologia.

[4] Lewis G.F., Carpentier A., Adeli K., Giacca A. (2002). Disordered fat storage and mobilization in the pathogenesis of insulin resistance and type 2 diabetes. Endocr. Rev..

[5] Unger R.H., Orci L. (2000). Lipotoxic diseases of nonadipose tissues in obesity. Int. J. Obes. Relat. Metab. Disord..

[6] Sun K., Kusminski C.M., Scherer P.E. (2011). Adipose tissue remodeling and obesity. J. Clin. Investig..

[7] Weisberg S.P., McCann D., Desai M., Rosenbaum M., Leibel R.L., Ferrante A.W. (2003). Obesity is associated with macrophage accumulation in adipose tissue. J. Clin. Investig..

[8] Wellen K.E., Hotamisligil G.S. (2003). Obesity-induced inflammatory changes in adipose tissue. J. Clin. Investig..

[9] Xu H., Barnes G.T., Yang Q., Tan G., Yang D., Chou C.J., Sole J., Nichols A., Ross J.S., Tartaglia L.A. (2003). Chronic inflammation in fat plays a crucial role in the development of obesity-related insulin resistance. J. Clin. Investig..

[10] Fabbrini E., Yoshino J., Yoshino M., Magkos F., Tiemann Luecking C., Samovski D., Fraterrigo G., Okunade A.L., Patterson B.W., Klein S. (2015). Metabolically normal obese people are protected from adverse effects following weight gain. J. Clin. Investig..

[11] Hodson L., Skeaff C.M., Fielding B.A. (2008). Fatty acid composition of adipose tissue and blood in humans and its use as a biomarker of dietary intake. Prog. Lipid Res..

[12] Marik P.E., Varon J. (2009). ω-3 Dietary supplements and the risk of cardiovascular events: A systematic review. Clin. Cardiol..

[13] Delarue J., LeFoll C., Corporeau C., Lucas D. (2004). *n*-3 Long chain polyunsaturated fatty acids: A nutritional tool to prevent insulin resistance associated to type 2 diabetes and obesity?. Reprod. Nutr. Dev..

[14] Buckley J.D., Howe P.R. (2010). Long-chain ω-3 polyunsaturated fatty acids may be beneficial for reducing obesity-a review. Nutrients.

[15] Flachs P., Rossmeisl M., Bryhn M., Kopecky J. (2009). Cellular and molecular effects of *n*-3 polyunsaturated fatty acids on adipose tissue biology and metabolism. Clin. Sci..

[16] Torres-Fuentes C., Schellekens H., Dinan T.G., Cryan J.F. (2015). A natural solution for obesity: Bioactives for the prevention and treatment of weight gain. A review. Nutr. Neurosci..

[17] Puglisi M.J., Hasty A.H., Saraswathi V. (2011). The role of adipose tissue in mediating the beneficial effects of dietary fish oil. J. Nutr. Biochem..

[18] Kalupahana N.S., Claycombe K.J., Moustaid-Moussa N. (2011). *n*-3 Fatty acids alleviate adipose tissue inflammation and insulin resistance: Mechanistic insights. Adv. Nutr..

[19] Pinel A., Morio-Liondore B., Capel F. (2014). *n*-3 Polyunsaturated fatty acids modulate metabolism of insulin-sensitive tissues: Implication for the prevention of type 2 diabetes. J. Physiol. Biochem..

[20] Martinez-Fernandez L., Laiglesia L.M., Huerta A.E., Martinez J.A., Moreno-Aliaga M.J. (2015). ω-3 Fatty acids and adipose tissue function in obesity and metabolic syndrome. Prostaglandins Other Lipid Mediat..

[21] Berge J.P., Barnathan G. (2005). Fatty acids from lipids of marine organisms: Molecular biodiversity, roles as biomarkers, biologically active compounds, and economical aspects. Adv. Biochem. Eng. Biotechnol..

[22] Rørå A.M.B., Ruyter B., Skorve J., Berge R., Slinning K.-E. (2005). Influence of high content of dietary soybean oil on quality of large fresh, smoked and frozen atlantic salmon (*Salmo salar*). Aquac. Int..

[23] Torstensen B.E., Bell J.G., Rosenlund G., Henderson R.J., Graff I.E., Tocher D.R., Lie O., Sargent J.R. (2005). Tailoring of atlantic salmon (*Salmo salar* L.) flesh lipid composition and sensory quality by replacing fish oil with a vegetable oil blend. J. Agric. Food Chem..

[24] Schuchardt J.P., Hahn A. (2013). Bioavailability of long-chain ω-3 fatty acids. Prostaglandins Leukot. Essent. Fat. Acids.

[25] Burdge G.C., Finnegan Y.E., Minihane A.M., Williams C.M., Wootton S.A. (2003). Effect of altered dietary *n*-3 fatty acid intake upon plasma lipid fatty acid composition, conversion of [^13^C]α-linolenic acid to longer-chain fatty acids and partitioning towards β-oxidation in older men. Br. J. Nutr..

[26] Bolton-Smith C., Woodward M., Tavendale R. (1997). Evidence for age-related differences in the fatty acid composition of human adipose tissue, independent of diet. Eur. J. Clin. Nutr..

[27] Ogura T., Takada H., Okuno M., Kitade H., Matsuura T., Kwon M., Arita S., Hamazaki K., Itomura M., Hamazaki T. (2010). Fatty acid composition of plasma, erythrocytes and adipose: Their correlations and effects of age and sex. Lipids.

[28] Walker C.G., Browning L.M., Mander A.P., Madden J., West A.L., Calder P.C., Jebb S.A. (2014). Age and sex differences in the incorporation of EPA and DHA into plasma fractions, cells and adipose tissue in humans. Br. J. Nutr..

[29] Browning L.M., Walker C.G., Mander A.P., West A.L., Madden J., Gambell J.M., Young S., Wang L., Jebb S.A., Calder P.C. (2012). Incorporation of eicosapentaenoic and docosahexaenoic acids into lipid pools when given as supplements providing doses equivalent to typical intakes of oily fish. Am. J. Clin. Nutr..

[30] Katan M.B., Deslypere J.P., van Birgelen A.P., Penders M., Zegwaard M. (1997). Kinetics of the incorporation of dietary fatty acids into serum cholesteryl esters, erythrocyte membranes, and adipose tissue: An 18-month controlled study. J. Lipid Res..

[31] Leaf D.A., Connor W.E., Barstad L., Sexton G. (1995). Incorporation of dietary *n*-3 fatty acids into the fatty acids of human adipose tissue and plasma lipid classes. Am. J. Clin. Nutr..

[32] Mostad I.L., Bjerve K.S., Bjorgaas M.R., Lydersen S., Grill V. (2006). Effects of *n*-3 fatty acids in subjects with type 2 diabetes: Reduction of insulin sensitivity and time-dependent alteration from carbohydrate to fat oxidation. Am. J. Clin. Nutr..

[33] Gammelmark A., Madsen T., Varming K., Lundbye-Christensen S., Schmidt E.B. (2012). Low-dose fish oil supplementation increases serum adiponectin without affecting inflammatory markers in overweight subjects. Nutr. Res..

[34] Witt P.M., Christensen J.H., Ewertz M., Aardestrup I.V., Schmidt E.B. (2010). The incorporation of marine *n*-3 PUFA into platelets and adipose tissue in pre- and postmenopausal women: A randomised, double-blind, placebo-controlled trial. Br. J. Nutr..

[35] McQuaid S.E., Humphreys S.M., Hodson L., Fielding B.A., Karpe F., Frayn K.N. (2010). Femoral adipose tissue may accumulate the fat that has been recycled as VLDL and nonesterified fatty acids. Diabetes.

[36] Kunesova M., Hlavaty P., Tvrzicka E., Stankova B., Kalouskova P., Viguerie N., Larsen T.M., van Baak M.A., Jebb S.A., Martinez J.A. (2012). Fatty acid composition of adipose tissue triglycerides after weight loss and weight maintenance: The diogenes study. Physiol. Res..

[37] Buckley J.D., Howe P.R. (2009). Anti-obesity effects of long-chain ω-3 polyunsaturated fatty acids. Obes. Rev..

[38] Du S., Jin J., Fang W., Su Q. (2015). Does fish oil have an anti-obesity effect in overweight/obese adults? A meta-analysis of randomized controlled trials. PLoS ONE.

[39] Sato T., Kameyama T., Ohori T., Matsuki A., Inoue H. (2014). Effects of eicosapentaenoic acid treatment on epicardial and abdominal visceral adipose tissue volumes in patients with coronary artery disease. J. Atheroscler. Thromb..

[40] Todorcevic M., Kjaer M.A., Djakovic N., Vegusdal A., Torstensen B.E., Ruyter B. (2009). *n*-3 HUFAs affect fat deposition, susceptibility to oxidative stress, and apoptosis in Atlantic salmon visceral adipose tissue. Comp. Biochem. Physiol. B Biochem. Mol. Biol..

[41] Ruzickova J., Rossmeisl M., Prazak T., Flachs P., Sponarova J., Veck M., Tvrzicka E., Bryhn M., Kopecky J. (2004). ω-3 PUFA of marine origin limit diet-induced obesity in mice by reducing cellularity of adipose tissue. Lipids.

[42] Camargo A., Meneses M.E., Perez-Martinez P., Delgado-Lista J., Jimenez-Gomez Y., Cruz-Teno C., Tinahones F.J., Paniagua J.A., Perez-Jimenez F., Roche H.M. (2014). Dietary fat differentially influences the lipids storage on the adipose tissue in metabolic syndrome patients. Eur. J. Nutr..

[43] Guebre-Egziabher F., Debard C., Drai J., Denis L., Pesenti S., Bienvenu J., Vidal H., Laville M., Fouque D. (2013). Differential dose effect of fish oil on inflammation and adipose tissue gene expression in chronic kidney disease patients. Nutrition.

[44] Itariu B.K., Zeyda M., Hochbrugger E.E., Neuhofer A., Prager G., Schindler K., Bohdjalian A., Mascher D., Vangala S., Schranz M. (2012). Long-chain *n*-3 pufas reduce adipose tissue and systemic inflammation in severely obese nondiabetic patients: A randomized controlled trial. Am. J. Clin. Nutr..

[45] Jimenez-Gomez Y., Cruz-Teno C., Rangel-Zuniga O.A., Peinado J.R., Perez-Martinez P., Delgado-Lista J., Garcia-Rios A., Camargo A., Vazquez-Martinez R., Ortega-Bellido M. (2014). Effect of dietary fat modification on subcutaneous white adipose tissue insulin sensitivity in patients with metabolic syndrome. Mol. Nutr. Food Res..

[46] Kratz M., Kuzma J.N., Hagman D.K., van Yserloo B., Matthys C.C., Callahan H.S., Weigle D.S. (2013). N3 PUFAs do not affect adipose tissue inflammation in overweight to moderately obese men and women. J. Nutr..

[47] Pena-Orihuela P., Camargo A., Rangel-Zuniga O.A., Perez-Martinez P., Cruz-Teno C., Delgado-Lista J., Yubero-Serrano E.M., Paniagua J.A., Tinahones F.J., Malagon M.M. (2013). Antioxidant system response is modified by dietary fat in adipose tissue of metabolic syndrome patients. J. Nutr. Biochem..

[48] Spencer M., Finlin B.S., Unal R., Zhu B., Morris A.J., Shipp L.R., Lee J., Walton R.G., Adu A., Erfani R. (2013). ω-3 Fatty acids reduce adipose tissue macrophages in human subjects with insulin resistance. Diabetes.

[49] Baillie R.A., Takada R., Nakamura M., Clarke S.D. (1999). Coordinate induction of peroxisomal acyl-coa oxidase and UCP-3 by dietary fish oil: A mechanism for decreased body fat deposition. Prostaglandins Leukot. Essent. Fat. Acids.

[50] Belzung F., Raclot T., Groscolas R. (1993). Fish oil *n*-3 fatty acids selectively limit the hypertrophy of abdominal fat depots in growing rats fed high-fat diets. Am. J. Physiol..

[51] Flachs P., Horakova O., Brauner P., Rossmeisl M., Pecina P., Franssen-van Hal N., Ruzickova J., Sponarova J., Drahota Z., Vlcek C. (2005). Polyunsaturated fatty acids of marine origin upregulate mitochondrial biogenesis and induce β-oxidation in white fat. Diabetologia.

[52] Hainault I., Carolotti M., Hajduch E., Guichard C., Lavau M. (1993). Fish oil in a high lard diet prevents obesity, hyperlipemia, and adipocyte insulin resistance in rats. Ann. N. Y. Acad. Sci..

[53] Oh D.Y., Talukdar S., Bae E.J., Imamura T., Morinaga H., Fan W., Li P., Lu W.J., Watkins S.M., Olefsky J.M. (2010). GPR120 is an ω-3 fatty acid receptor mediating potent anti-inflammatory and insulin-sensitizing effects. Cell.

[54] Perez-Matute P., Perez-Echarri N., Martinez J.A., Marti A., Moreno-Aliaga M.J. (2007). Eicosapentaenoic acid actions on adiposity and insulin resistance in control and high-fat-fed rats: Role of apoptosis, adiponectin and tumour necrosis factor-α. Br. J. Nutr..

[55] Saraswathi V., Gao L., Morrow J.D., Chait A., Niswender K.D., Hasty A.H. (2007). Fish oil increases cholesterol storage in white adipose tissue with concomitant decreases in inflammation, hepatic steatosis, and atherosclerosis in mice. J. Nutr..

[56] Barber E., Sinclair A.J., Cameron-Smith D. (2013). Comparative actions of ω-3 fatty acids on *in-vitro* lipid droplet formation. Prostaglandins Leukot. Essent. Fat. Acids.

[57] DeClercq V., d’Eon B., McLeod R.S. (2015). Fatty acids increase adiponectin secretion through both classical and exosome pathways. Biochim. Biophys. Acta.

[58] Guo W., Xie W., Lei T., Hamilton J.A. (2005). Eicosapentaenoic acid, but not oleic acid, stimulates β-oxidation in adipocytes. Lipids.

[59] Kim H.K., Della-Fera M., Lin J., Baile C.A. (2006). Docosahexaenoic acid inhibits adipocyte differentiation and induces apoptosis in 3T3-L1 preadipocytes. J. Nutr..

[60] Kusunoki C., Yang L., Yoshizaki T., Nakagawa F., Ishikado A., Kondo M., Morino K., Sekine O., Ugi S., Nishio Y. (2013). ω-3 Polyunsaturated fatty acid has an anti-oxidant effect via the Nrf-2/HO-1 pathway in 3T3-L1 adipocytes. Biochem. Biophys. Res. Commun..

[61] Lorente-Cebrian S., Bustos M., Marti A., Martinez J.A., Moreno-Aliaga M.J. (2010). Eicosapentaenoic acid up-regulates apelin secretion and gene expression in 3T3-L1 adipocytes. Mol. Nutr. Food Res..

[62] Murali G., Desouza C.V., Clevenger M.E., Ramalingam R., Saraswathi V. (2014). Differential effects of eicosapentaenoic acid and docosahexaenoic acid in promoting the differentiation of 3T3-L1 preadipocytes. Prostaglandins Leukot. Essent. Fat. Acids.

[63] Murumalla R.K., Gunasekaran M.K., Padhan J.K., Bencharif K., Gence L., Festy F., Cesari M., Roche R., Hoareau L. (2012). Fatty acids do not pay the toll: Effect of SFA and PUFA on human adipose tissue and mature adipocytes inflammation. Lipids Health Dis..

[64] Oster R.T., Tishinsky J.M., Yuan Z., Robinson L.E. (2010). Docosahexaenoic acid increases cellular adiponectin mRNA and secreted adiponectin protein, as well as PPARγ mRNA, in 3T3-L1 adipocytes. Appl. Physiol. Nutr. Metab..

[65] Prostek A., Gajewska M., Kamola D., Balasinska B. (2014). The influence of EPA and DHA on markers of inflammation in 3T3-L1 cells at different stages of cellular maturation. Lipids Health Dis..

[66] Tishinsky J.M., Ma D.W., Robinson L.E. (2011). Eicosapentaenoic acid and rosiglitazone increase adiponectin in an additive and PPARγ-dependent manner in human adipocytes. Obesity.

[67] Vaidya H., Cheema S.K. (2015). Arachidonic acid has a dominant effect to regulate lipogenic genes in 3T3-L1 adipocytes compared to ω-3 fatty acids. Food Nutr. Res..

[68] Wang Y.C., Kuo W.H., Chen C.Y., Lin H.Y., Wu H.T., Liu B.H., Chen C.H., Mersmann H.J., Chang K.J., Ding S.T. (2010). Docosahexaenoic acid regulates serum amyloid a protein to promote lipolysis through down regulation of perilipin. J. Nutr. Biochem..

[69] Wojcik C., Lohe K., Kuang C., Xiao Y., Jouni Z., Poels E. (2014). Modulation of adipocyte differentiation by ω-3 polyunsaturated fatty acids involves the ubiquitin-proteasome system. J. Cell. Mol. Med..

[70] Hodson L., Humphreys S.M., Karpe F., Frayn K.N. (2013). Metabolic signatures of human adipose tissue hypoxia in obesity. Diabetes.

[71] Ji H., Li J., Liu P. (2011). Regulation of growth performance and lipid metabolism by dietary *n*-3 highly unsaturated fatty acids in juvenile grass carp, ctenopharyngodon idellus. Comp. Biochem. Physiol. B Biochem. Mol. Biol..

[72] Liu P., Li C., Huang J., Ji H. (2014). Regulation of adipocytes lipolysis by *n*-3 HUFA in grass carp (*Ctenopharyngodon idellus*) *in vitro* and *in vivo*. Fish. Physiol. Biochem..

[73] Desai M., Beall M., Ross M.G. (2013). Developmental origins of obesity: Programmed adipogenesis. Curr. Diab. Rep..

[74] McMillen I.C., Robinson J.S. (2005). Developmental origins of the metabolic syndrome: Prediction, plasticity, and programming. Physiol. Rev..

[75] Catalano P.M., Ehrenberg H.M. (2006). The short- and long-term implications of maternal obesity on the mother and her offspring. BJOG.

[76] Sewell M.F., Huston-Presley L., Super D.M., Catalano P. (2006). Increased neonatal fat mass, not lean body mass, is associated with maternal obesity. Am. J. Obstet. Gynecol..

[77] Muhlhausler B.S., Gibson R.A., Makrides M. (2011). The effect of maternal ω-3 long-chain polyunsaturated fatty acid (*n*-3 LCPUFA) supplementation during pregnancy and/or lactation on body fat mass in the offspring: A systematic review of animal studies. Prostaglandins Leukot. Essent. Fat. Acids.

[78] Muhlhausler B.S., Miljkovic D., Fong L., Xian C.J., Duthoit E., Gibson R.A. (2011). Maternal ω-3 supplementation increases fat mass in male and female rat offspring. Front. Genet..

[79] Hilton C., Karpe F., Pinnick K.E. (2015). Role of developmental transcription factors in white, brown and beige adipose tissues. Biochim. Biophys. Acta.

[80] Pinnick K.E., Neville M.J., Fielding B.A., Frayn K.N., Karpe F., Hodson L. (2012). Gluteofemoral adipose tissue plays a major role in production of the lipokine palmitoleate in humans. Diabetes.

[81] Pinnick K.E., Nicholson G., Manolopoulos K.N., McQuaid S.E., Valet P., Frayn K.N., Denton N., Min J.L., Zondervan K.T., Fleckner J. (2014). Distinct developmental profile of lower-body adipose tissue defines resistance against obesity-associated metabolic complications. Diabetes.

[82] Lee M.S., Kwun I.S., Kim Y. (2008). Eicosapentaenoic acid increases lipolysis through up-regulation of the lipolytic gene expression and down-regulation of the adipogenic gene expression in 3T3-L1 adipocytes. Genes Nutr..

[83] Todorcevic M., Vegusdal A., Gjoen T., Sundvold H., Torstensen B.E., Kjaer M.A., Ruyter B. (2008). Changes in fatty acids metabolism during differentiation of atlantic salmon preadipocytes; effects of *n*-3 and *n*-9 fatty acids. Biochim. Biophys. Acta.

[84] Wendel M., Heller A.R. (2009). Anticancer actions of ω-3 fatty acids–Current state and future perspectives. Anticancer Agents Med. Chem..

[85] Prins J.B., Walker N.I., Winterford C.M., Cameron D.P. (1994). Apoptosis of human adipocytes in vitro. Biochem. Biophys. Res. Commun..

[86] Prins J.B., O'Rahilly S. (1997). Regulation of adipose cell number in man. Clin. Sci..

[87] Nelson-Dooley C., Della-Fera M.A., Hamrick M., Baile C.A. (2005). Novel treatments for obesity and osteoporosis: Targeting apoptotic pathways in adipocytes. Curr. Med. Chem..

[88] Wyllie A.H. (1997). Apoptosis: An overview. Br. Med. Bull..

[89] Tocher D.R. (2003). Metabolism and functions of lipids and fatty acids in teleost fish. Rev. Fish. Sci..

[90] Abele D., Puntarulo S. (2004). Formation of reactive species and induction of antioxidant defence systems in polar and temperate marine invertebrates and fish. Comp. Biochem. Physiol. A Mol. Integr. Physiol..

[91] Biondo P.D., Brindley D.N., Sawyer M.B., Field C.J. (2008). The potential for treatment with dietary long-chain polyunsaturated *n*-3 fatty acids during chemotherapy. J. Nutr. Biochem..

[92] Calviello G., Palozza P., Maggiano N., Piccioni E., Franceschelli P., Frattucci A., Di Nicuolo F., Bartoli G.M. (1999). Cell proliferation, differentiation, and apoptosis are modified by *n*-3 polyunsaturated fatty acids in normal colonic mucosa. Lipids.

[93] Todorcevic M., Skugor S., Ruyter B. (2010). Alterations in oxidative stress status modulate terminal differentiation in atlantic salmon adipocytes cultivated in media rich in *n*-3 fatty acids. Comp. Biochem. Physiol. B Biochem. Mol. Biol..

[94] Maassen J.A., Romijn J.A., Heine R.J. (2008). Fatty acid-induced mitochondrial uncoupling in adipocytes as a key protective factor against insulin resistance and β cell dysfunction: Do adipocytes consume sufficient amounts of oxygen to oxidise fatty acids?. Diabetologia.

[95] Bouwman F.G., Wang P., van Baak M., Saris W.H., Mariman E.C. (2014). Increased β-oxidation with improved glucose uptake capacity in adipose tissue from obese after weight loss and maintenance. Obesity.

[96] Couet C., Delarue J., Ritz P., Antoine J.M., Lamisse F. (1997). Effect of dietary fish oil on body fat mass and basal fat oxidation in healthy adults. Int. J. Obes. Relat. Metab. Disord..

[97] Lorente-Cebrian S., Bustos M., Marti A., Martinez J.A., Moreno-Aliaga M.J. (2009). Eicosapentaenoic acid stimulates amp-activated protein kinase and increases visfatin secretion in cultured murine adipocytes. Clin. Sci..

[98] Flachs P., Rossmeisl M., Kuda O., Kopecky J. (2013). Stimulation of mitochondrial oxidative capacity in white fat independent of UCP1: A key to lean phenotype. Biochim. Biophys. Acta.

[99] Zhao M., Chen X. (2014). Eicosapentaenoic acid promotes thermogenic and fatty acid storage capacity in mouse subcutaneous adipocytes. Biochem. Biophys. Res. Commun..

[100] Hosogai N., Fukuhara A., Oshima K., Miyata Y., Tanaka S., Segawa K., Furukawa S., Tochino Y., Komuro R., Matsuda M. (2007). Adipose tissue hypoxia in obesity and its impact on adipocytokine dysregulation. Diabetes.

[101] Trayhurn P., Wood I.S. (2004). Adipokines: Inflammation and the pleiotropic role of white adipose tissue. Br. J. Nutr..

[102] Wood I.S., de Heredia F.P., Wang B., Trayhurn P. (2009). Cellular hypoxia and adipose tissue dysfunction in obesity. Proc. Nutr. Soc..

[103] Ye J., Gao Z., Yin J., He Q. (2007). Hypoxia is a potential risk factor for chronic inflammation and adiponectin reduction in adipose tissue of *ob*/*ob* and dietary obese mice. Am. J. Physiol. Endocrinol. Metab..

[104] Todoric J., Loffler M., Huber J., Bilban M., Reimers M., Kadl A., Zeyda M., Waldhausl W., Stulnig T.M. (2006). Adipose tissue inflammation induced by high-fat diet in obese diabetic mice is prevented by *n*-3 polyunsaturated fatty acids. Diabetologia.

[105] Fantuzzi G. (2008). Adiponectin and inflammation: Consensus and controversy. J. Allergy Clin. Immunol..

[106] Romacho T., Glosse P., Richter I., Elsen M., Schoemaker M.H., van Tol E.A., Eckel J. (2015). Nutritional ingredients modulate adipokine secretion and inflammation in human primary adipocytes. Nutrients.

[107] Lorente-Cebrian S., Perez-Matute P., Martinez J.A., Marti A., Moreno-Aliaga M.J. (2006). Effects of eicosapentaenoic acid (EPA) on adiponectin gene expression and secretion in primary cultured rat adipocytes. J. Physiol. Biochem..

[108] Murata M., Kaji H., Takahashi Y., Iida K., Mizuno I., Okimura Y., Abe H., Chihara K. (2000). Stimulation by eicosapentaenoic acids of leptin mrna expression and its secretion in mouse 3T3-L1 adipocytes *in vitro*. Biochem. Biophys. Res. Commun..

[109] Perez-Matute P., Marti A., Martinez J.A., Fernandez-Otero M.P., Stanhope K.L., Havel P.J., Moreno-Aliaga M.J. (2005). Eicosapentaenoic fatty acid increases leptin secretion from primary cultured rat adipocytes: Role of glucose metabolism. Am. J. Physiol. Regul. Integr. Comp. Physiol..

[110] Reseland J.E., Haugen F., Hollung K., Solvoll K., Halvorsen B., Brude I.R., Nenseter M.S., Christiansen E.N., Drevon C.A. (2001). Reduction of leptin gene expression by dietary polyunsaturated fatty acids. J. Lipid Res..

[111] Aranceta J., Perez-Rodrigo C. (2012). Recommended dietary reference intakes, nutritional goals and dietary guidelines for fat and fatty acids: A systematic review. Br. J. Nutr..

